# Impact of COVID-19 in the Perioperative Period of Cardiovascular Surgery

**DOI:** 10.21470/1678-9741-2021-0611

**Published:** 2022

**Authors:** Adnaldo da Silveira Maia

**Affiliations:** 1 Instituto Dante Pazzanese de Cardiologia, IDPC, São Paulo, Brazil

There is great interest from the scientific community in determining the safe period for approaching patients who need cardiovascular surgery in the context of the current pandemic, as well as the complications inherent to COVID-19 infection. The work produced by Gomes et al.^[1]^, “COVID-19 in the Perioperative Period of Cardiovascular Surgery: the Brazilian Experience”, a multicentric and retrospective analysis of 104 patients, clearly describes the impact of this infection on patients undergoing cardiovascular surgery.

The division between groups using the surgery-infection time relationship allows us to delineate the outcomes presented. The high mortality identified in groups 2 and 3 corroborates the data described in the literature. In addition to this finding, the prevalence of acute renal failure is highlighted, as well as the longer hospital stay between the groups. The changes and protocols initiated by several surgical services for the care of COVID-19 patients were important in the pandemic, as described by Casanova et al.^[2]^, with the objective of reducing the spreading of the infection and its inherent complications.

Yates et al.^[3]^, in a descriptive analysis of 97 patients in which 7 of these individuals were diagnosed with COVID-19 infection in the immediate postoperative period, underwent the following procedures: coronary artery bypass grafting (CABG, n=4), aortic valve replacement (n=1), aortic valve replacement + CABG (n=1) and mitral valve replacement + CABG (n=1). The mortality rate in this case series was 3 (43%) patients. The authors also emphasize that all 3 deaths were due to respiratory failure refractory to the measures adopted by the assistant team.

In another case series, Farsky et al.^[4]^, in a single-center analysis of 13 cases undergoing CABG surgery, identified 3 deaths. Patients with a positive PCR testing for SARS-CoV-2 before surgery waited 14 days for symptoms to resolve according to institutional protocol. Two of these deaths were due to septic shock and encephalopathy. Three patients underwent emergency CABG, therefore with unavailable previous exams, of which 1 patient evolved with cardiogenic shock and death on the 3^rd^ postoperative day. In addition to these findings, the patients had a long hospital stay as well as other complications, such as renal failure and pulmonary thromboembolism.

Such studies corroborate the findings of Gomes et al.^[1]^ that there is high mortality and morbidity associated with COVID-19 infection in patients undergoing cardiovascular surgery Longer hospital stay, pulmonary complications, renal failure and thromboembolic events are present in these patients. Preoperative management protocols and increasingly robust scientific data to understand the evolution of patients between different times of infection are the direction in this time of uncertainty.
